# *Helicobacter Pylori* Detected in Tap Water of Peruvian Patients with Gastric Cancer

**DOI:** 10.31557/APJCP.2019.20.11.3193

**Published:** 2019

**Authors:** Miluska Castillo, Luis A Bernabe, Carlos A Castaneda, Ivan Chavez, Eloy Ruiz, Fernando Barreda, Daniel Valdivia, Nancy Suarez, Jais Nieves, Emmanuel Dias-Neto, Kevin F Boehnke, Maria P Landa-Baella, Paola Montenegro

**Affiliations:** 1 *Instituto Nacional de Enfermedades Neoplasicas, Lima, *; 2 *Universidad Científica del Sur, Peru, *; 3 *CIPE, A.C.Camargo Cancer Center, São Paulo, Brazil, *; 4 *University of Michigan, Ann Arbor, Michigan, USA. *

**Keywords:** Helicobacter pylori, cancer gastric, tap-water, urea, hspA

## Abstract

**Objective:**

To evaluate the correlation between the presence of *H. pylori *in paired samples of tap water and gastric cancer (GC) lesion in Lima city (Peru).

**Material and methods:**

Gastric tissue and tap-water samples were prospectively collected from 82 Gastric Cancer who lived in Lima. HspA and ureA genes were evaluated by qPCR in the samples.

**Results:**

The median age of patients with GC was 63 years, 52.4% were men and stage-II in 36.6%. A home-living time> 10 years was reported in 84.1% of patients. Boiling water treatment was indicated in 85.4% of cases. *H. pylori *was detected in 69.5% of gastric tissues and in 12.2% of analyzed tap-water. There was no differences in gastric infection rates among those with or without water contamination (70% vs. 69.4%, p=0.971).

**Conclusion & Impact:**

*H. pylori *was found in tap-water samples, however, detection rates were lower than in gastric cancer samples. Other sources of infection transmission should be investigated.

## Introduction

Gastric cancer (GC) is one of the most common cancers worldwide and is the second most common in Peru (https://goo.gl/KU1jzK). The critical role of chronic *Helicobacter pylori* (*H. pylori*) infection has been demonstrated in the development of intestinal metaplasia and gastric malignancy (Talley et al., 2008). The prevalence of 20% *H. pylori* infection among adolescents in the United States pales in comparison to infection rates that exceed 90% at 5 years of age in developing countries (Klein et al., 1991). Some studies have pointed out the role of poor sanitation in the spread of this organism because *H. pylori* has been found in the feces of children living in an endemic area (Thomas et al., 1992). *H. pylori* can survive in water for prolonged periods (Enroth and Engstrand, 1995), and *H. pylori* have been described in drinking water through polymerase chain reaction (PCR), suggesting that water can be an important reservoir for *H. pylori* infection (Hulten et al., 1998; Boehnke et al., 2018). A higher positivity rate of *H. pylori* contamination in drinking water has been implicated as one of the causes of the high GC rates observed in some countries (Boehnke et al., 2018; Klein et al., 1991). However, we do not know of any previous study that has detected *H. pylori* in paired samples of gastric tissue and drinking water of patients with GC.

This study will detect the presence of *H. pylori* in tap water of Peruvian patients with GC and assess whether water contamination is correlated with the presence of *H. Pylori* in gastric samples.

## Materials and Methods

*Study population*

Observational cohort study in prospectively recruited patients with a recent diagnosis of GC (n= 82 patients). All patients underwent gastroscopy or surgery at the Instituto Nacional de Enfermedades Neoplasicas (INEN) between February 2014 and February 2017. Inclusion criteria included the histology of gastric adenocarcinoma and domicile in Lima city. Participants completed questionnaires with information on home location and epidemiological characteristics. The pathological clinical data were obtained from the patient file and the pathological report.

This study and the informed consent were approved by the Ethics Committee of the INEN (010-2015-CRP-DI-DICON / INEN15-10).

*Sample collection and processing*

The visits to the patients’ homes were organized by Lima sectors (Castaneda et al., 2019), coordinated by phone under the supervision of two scientists (MC and LB) and water samples were collected from the kitchen faucet. A total of 2L was collected in sterile bottles per-home and transported at room temperature to the laboratory within an approximate range of 2 hours. The samples were preprocessed through a 0.10 μm pore polyether sulfone membrane filter (Stericup, Millipore, MA, USA) in a vacuum filter system. The membranes were placed in Petri dishes and stored at -20°C until further processing. To optimize sample collection, the membranes were crushed into small fragments and a solution of PBS+tween 20 (0.2%) was added and centrifuged at 7500 g for 10 minutes. The supernatant was discarded and then TE 1x buffer was added. Then, it was centrifuged again at 7,500g for 10 minutes and the supernatant was discarded once more as previously described (8). The sediment was processed using the GenEluteTM BacterialGenomic DNA (SIGMA) kit, according to the manufacturer’s instructions. Total DNA concentration was measured using fluorometric quantification using the QubitdsDNA HS test kit (Thermo Fisher Scientific, Lithuania). The extracted DNA was used for subsequent qPCR experiments. The detection of *H. pylori* was carried out using the quantitative PCR method (qPCR) under the supervision of 2 scientists (JN and NS) according to what has been previously described in a publication of our group (Castaneda et al., 2019). For the water samples, from a technical point of view, the detection limit of 0.2 copies/μl of the *H. pylori* genome was determined as positive for contamination, assuming that bacteria or fragments can be found in the water sample of these genetic material. The infection of the gastric tissue and the state of contamination of the water samples were determined as positive with at least one result of the ureA / hspA gene.

*Statistic analysis*

Summary descriptive statistics and graphic screens were generated. The Pearson Chi-Square test and Fisher’s Statistical Test were performed to demonstrate the hypothesis that *H. pylori* contamination in water is correlated with a higher rate of stomach infection. Statistical analysis was performed with the SPSS statistical software package for Windows (Version25.0.0, SPSS Inc., Chicago, IL, United States). Research procedures and analysis are supported by the STROBE Guidelines.

## Results

Median age was 63 years and most patients were male (52.4%). Fifty-nine (72%) patients had a birthplace out of Lima city. Most patients were residents in south (22%), north (18.3%), east (20.7%), and center (18.3%) of Lima sector or Callao (11%). Most patients had a home-living time>10 years (84.1%) and had a drinking water supply network (93.9%). Boiling water treatment was indicated in 85.4% of cases. 

Tumor location was more frequent in antrum (61%), and most frequent histology were diffuse (47.8%), intestinal (36.2%) or mixed (15.9%). Histology grade 3 and 2 were found in 51.2% and 25.6%, respectively. Clinical stage was II in 36.6%, III in 34.1% and IV in 12.2%. Additional pathological finding was intestinal metaplasia in 36.6%, chronic gastritis in 23.2%, dysplasia in 3.7% and intestinal polyp in 2.4%. *H. pylori* was detected in 69.5% (57/82) of the analyzed gastric tissues.

*H. pylori* genes were detected in 7 water samples with values above the detection limit of 0.2 copies of the *H. pylori* genome and 3 samples with values below this limit. The total of 10/82 samples (12.2%) were included to increase the level of confidence. Detection of hspA gene alone was found in 40% (4/10), ureA gene alone in 80% (8/10) and both genes in 20% (2/10). The median hspA value was 0.02 copies / µl (0 - 124.38) and the ureA was 3.15 copies / µl (0 - 122.91). Positive samples for *H. pylori* were found in the 3 sectors of Lima. Samples of contaminated water came from the Santa Anita (1/10), San Juan de Lurigancho (2/10) and La Molina (1/16) (East), Rimac (1/10) (Downtown) districts, Chorrillos (1/10), Villa El Salvador (1/10) and Pachacamac (1/10) (Southern sector), there were 2 contaminated samples in Callao and one of them reached the highest values (hspA: 124.38 copies and ureA: 122.91 copies).

No differences were found between the rate of *H. pylori* infection in gastric tissue of cases that presented contamination in tap water versus those that did not (70% vs. 69.4%, p= 0.971).

## Discussion

This study is the first to investigate the presence of *H. pylori* in tap water of patients with GC who live in the city of Lima and detected contamination in >10% of the samples. Although, tap water is a hostile environment for the growth of microorganisms due to low nutritional levels and the presence of disinfection substances, our study reinforces the argument of a possible transmission route through this medium (Johnson et al., 1997). The rate we found differs from an earlier study that evaluated the expression of *H. pylori* genes by PCR and found higher contamination rates (50%) in 48 samples of drinking water from a very poor and new district of Lima (Hulten et al., 1996), but closer to a more recent evaluation (20%) in different Lima districts (Boehnke et al., 2018).

The evaluation of the presence of *H. pylori* through the PCR technique in tap water has also been studied in different continents; however, details about the methodology such as liters of analyzed water and the evaluation of the biofilm differ from each other and cause difficulties in interpretation. Studies in countries such as Pakistan, Iran, England and Mexico evaluate the presence of *H. pylori* in less than 270 samples of drinking water and found contamination rates of up to 40% (Amirhooshang et al., 2014; Ahmed et al., 2006). Mazari-Hiriart et al., (2001) evaluated the *H. pylori* 16S RNA gene in 139 water samples from five water systems in the Mexico City area and found 58 (42%) positive samples in total, however there were large differences in pollution rates between systems of water treatment (0% in the water supply system No. 3 vs 100% in the system No. 2). In addition, some of the mentioned studies have also evaluated the viability of *H. pylori* in drinking water through cultures and found viable agents only in a small fraction of the samples (Horiuchi et al., 2001; Watson et al., 2004). Non-culturable cocoid form of *H. pylori* has also been described in the water (Cellini et al., 1994; West et al., 1990).

We found an absence of correlation between the presence of *H. pylori* in tap-water and in gastric tissue, which could be related to the high infection rate of our GC patients (69.5%), the use of antibiotics in previously infected patients and because tap-water route of contamination would not be the most important in our environment.

The questionnaires in our research indicate that the custom of boiling tap water before drinking extends to the majority of our population, and it could eliminate living bacteria from the water. Johnson et al. evaluated the effect of chlorination on three strains of *H. pylori* and found that the organisms were inactivated by the levels of chlorine below that commonly used to treat drinking water, although it could have a greater tolerance than Escherichia coli (Johnson et al., 1997). Ahmed et al., (2006) reported that 71% of people who consume municipal water acquired *H. pylori* compared to 12% of people who consumed boiled or filtered water. They also found that subjects who preferred homemade food (57%) showed a lower prevalence of *H. pylori* than those who used to eat outside (80%).

Finally, we must indicate that *H. pylori* infection is an established risk factor for GC but factors such as dietary patterns and consumption of salt, tobacco and possibly environmental and occupational exposure may modulate the carcinogenic activity of *H. pylori* (Talley et al., 2008).

Among the limitations of our study is the small number of samples examined for a city as large as Lima, which comprises 43 districts and has an estimated population of >9 million people (https://goo.gl/KU1jzK). This cross-sectional study evaluated the *H. pylori* DNA in water only once. The variation in water quality during an annual or greater period was not evaluated. 

In conclusion, the results of this study support the hypothesis that *H. pylori* is in the environment of GC patients, such as tap water from their homes., However, the higher rates of infection in gastric tumor samples than the rates of contamination in water samples indicate that there are additional infectious sources.
